# Epidemiological profile of urinary tract infections in children before and during the COVID-19 pandemic: an observational study

**DOI:** 10.1590/1984-0462/2026/44/2025365

**Published:** 2026-09-07

**Authors:** Bruna Luiza Thesolim, Bárbara Martins Lima, Cristina Ortiz Sobrinho Valete

**Affiliations:** a Universidade Federal de São Carlos, São Carlos, SP, Brazil.; b Hospital Universitário da Universidade Federal de São Carlos, São Carlos, SP, Brazil.

**Keywords:** Urinary tract infections, Pediatric, COVID-19, Epidemiology, Microbial sensitivity tests, Infecções do trato urinário, Pediatria, COVID-19, Epidemiologia, Testes de sensibilidade microbiana

## Abstract

**Objective:**

The aim of this study was to evaluate whether the COVID-19 pandemic influenced the epidemiological, microbiological, and antimicrobial resistance profiles of urinary tract infections (UTI) in children admitted to a pediatric service.

**Methods:**

This retrospective ecological study included patients under 12 years of age admitted to a Brazilian university hospital with a diagnosis of UTI based on clinical and laboratory criteria. Two periods were analyzed: 2018-2019 (pre-pandemic) and 2022-2023 (pandemic period).

**Results:**

A total of 8007 urine cultures were analyzed, of which 203 met criteria for UTI (128 in the first and 75 in the second period). A higher proportion of neonates was observed in the second period. *Escherichia coli* remained the predominant pathogen. *Klebsiella pneumoniae* showed an increase in frequency (p=0.02) and resistance (p<0.001), while *Pseudomonas aeruginosa* also increased in frequency (p=0.02) and resistance (p=0.01). The susceptibility profile of other pathogens remained stable, with persistently high sensitivity to amikacin, meropenem, and third-generation cephalosporins.

**Conclusions:**

The COVID-19 pandemic period was associated with changes in the epidemiological and microbiological profile of pediatric UTIs. The increased frequency of healthcare-associated pathogens highlights the importance of continuous microbiological surveillance and rational antimicrobial use in pediatric practice.

## RESUMO

**Objetivo:**

O objetivo deste estudo foi avaliar se a pandemia de COVID-19 influenciou os perfis epidemiológico, microbiológico e de resistência antimicrobiana das infecções do trato urinário (ITU) em crianças atendidas em um serviço pediátrico.

**Métodos:**

Este estudo retrospectivo ecológico incluiu pacientes menores de 12 anos internados em um hospital universitário brasileiro com diagnóstico de ITU baseado em critérios clínicos e laboratoriais. Foram analisados dois períodos: 2018-2019 (pré-pandemia) e 2022-2023 (período pandêmico).

**Resultados:**

Foram analisadas 8007 uroculturas, das quais 203 atenderam aos critérios para ITU (128 no primeiro e 75 no segundo período). Observou-se maior proporção de recém-nascidos no segundo período. *Escherichia coli* permaneceu como o principal patógeno. *Klebsiella pneumoniae* apresentou aumento na frequência (p=0,02) e na resistência (p<0,001), enquanto *Pseudomonas aeruginosa* também aumentou em frequência (p=0,02) e resistência (p=0,01). O perfil de sensibilidade dos demais patógenos permaneceu estável, com altas taxas de sensibilidade à amicacina, meropenem e cefalosporinas de terceira geração.

**Conclusões:**

O período da pandemia de COVID-19 foi associado a mudanças no perfil epidemiológico e microbiológico das ITU pediátricas. O aumento na frequência de patógenos associados à assistência à saúde ressalta a importância da vigilância microbiológica contínua e do uso racional de antimicrobianos na prática pediátrica.

## INTRODUCTION

Urinary tract infections (UTIs) are among the most frequent causes of bacterial infection during childhood, accounting for up to 8% of the pediatric population, and are a leading cause of emergency room consulting and hospital admission.^1^ ^,^ ^2^ Also, UTIs can complicate into pyelonephritis, permanent kidney scarring, high blood pressure, and, in more severe cases, chronic kidney failure, especially when diagnosis and treatment are not effectively instituted.^2^

*Escherichia coli (E. coli)* is the leading pathogen in 70-90% of community UTI cases. However, pathogens such as *Klebsiella pneumoniae (K. pneumoniae)*, *Proteus mirabilis (P. mirabilis)*, and *Pseudomonas aeruginosa* (*P. aeruginosa*) appear in greater proportions in hospital settings, in children with comorbidities, or in the presence of predisposing factors, such as structural anomalies of the urinary tract and the use of urinary catheters. This more diverse etiological spectrum is usually associated with complicated conditions, with a greater risk of antimicrobial resistance.^3^

The gold standard for UTI diagnosis is a positive urine culture, defined by the detection of ≥100,000 colony-forming units per milliliter (CFU/mL) in midstream urine or ≥50,000 CFU/mL in catheter collection, associated with the presence of pyuria. Sample collection varies according to age and clinical condition and may be performed by collection bag, bladder catheterization, or suprapubic aspiration in selected cases. Despite the time required for culture results (24-72 h), initiation of empirical antimicrobial treatment should not be delayed, especially in cases with high fever or systemic compromise.^4^

The initial management of pediatric UTI is often empirical, supported by clinical protocols and international guidelines. In these circumstances, up-to-date knowledge of local microbiological profiles is crucial to guide effective therapeutic choices and reduce treatment failures. Continuous microbiological surveillance is also essential to monitor trends in antimicrobial resistance, a growing global problem, and is considered one of the greatest threats to contemporary public health.^5^

The COVID-19 pandemic has introduced significant changes in pediatric care practices, including fewer routine appointments, fewer diagnostic tests used, and increased empirical antimicrobial prescriptions. These changes may have altered the epidemiology of UTIs, either by reducing the detection of mild cases or by selecting more resistant pathogens in hospital settings. Recent studies suggest that this period contributed to accelerating previously observed resistance trends and altered patterns of medical care seeking.^6^ ^,^ ^7^ ^,^ ^8^

In this context, understanding the pandemic’s impact on the epidemiology and resistance dynamics of pediatric UTIs is essential. Therefore, the aim of this study was to evaluate whether the COVID-19 pandemic influenced the epidemiological, microbiological, and antimicrobial profiles of urinary tract infections in children admitted to a pediatric service.

## METHOD

This retrospective ecological study was conducted at the University Hospital of the Federal University of São Carlos, a public tertiary teaching hospital located in the state of São Paulo, Brazil. The hospital provides pediatric care through a referral-based Emergency Department, receiving patients from primary healthcare units, urgent care units, and pre-hospital emergency services, covering a broad spectrum of low- to high-complexity cases.

Data were obtained from microbiology laboratory records provided by the Hospital Infection Control Service, including all urine cultures processed between January 2018 and December 2019 (pre-pandemic period) and between January 2022 and December 2023 (pandemic period). The years 2020 and 2021 were excluded due to the profound disruptions in healthcare delivery during the peak of the COVID-19 pandemic, including changes in healthcare-seeking behavior, prioritization of urgent care, and modifications in diagnostic and laboratory practices. These factors may affect the comparability between periods and should be considered when interpreting the results. Additionally, these years were considered a transitional period, not representative of stable epidemiological patterns.

Medical records of patients with positive urine cultures were reviewed to identify clinical and laboratory criteria consistent with UTI. Only patients who were admitted to the hospital after evaluation in the Emergency Department were included in the analysis. Inclusion criteria were patients aged under 12 years who presented with at least one compatible clinical symptom (fever, irritability, prostration, dysuria, pollakiuria, abdominal pain, back pain, or hematuria) associated with significant bacterial growth in urine culture (≥10^5^ CFU/mL in midstream urine or catheterized samples). Urine samples collected using adhesive urine bags were excluded due to their known high contamination rates. Additional exclusion criteria included incomplete medical records, inconclusive cultures, and polymicrobial growth. Recurrent UTI episodes in the same patient were considered as independent events when fulfilling the predefined clinical and laboratory criteria.

Data were extracted using a structured Excel data collection form developed by the researchers, including the following variables: age, sex, month of diagnosis, isolated microorganism, and antimicrobial susceptibility profile. Age was categorized according to traditional pediatric age groups as follows: neonates up to 28 days of life, infants aged 29 days to 2 years, preschool children aged 2-7 years, and school-aged children aged 7-11 years. Patients were categorized into two groups according to the period of diagnosis: pre-pandemic (2018-2019) and pandemic period (2022-2023).

The study was approved by the institutional Research Ethics Committee, with exemption from informed consent due to its retrospective nature. This article was prepared in accordance with the Strengthening the Reporting of Observational Studies in Epidemiology (STROBE) statement.^9^

Statistical analyses were performed using Stata version 19.0 (StataCorp, College Station, Texas, USA). Categorical variables were expressed as frequencies and percentages and compared using the chi-square test or Fisher’s exact test, as appropriate. Statistical significance was defined as p<0.05.

## RESULTS

**Figure 1.**
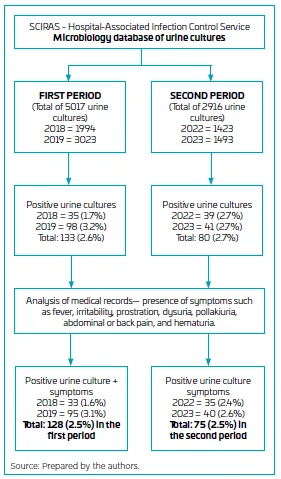
Flowchart of urine culture selection and inclusion of pediatric patients with confirmed urinary tract infection in a tertiary hospital setting, based on clinical and laboratory criteria.

Between 2018 and 2019, a total of 5017 urine cultures were performed (1994 in 2018 and 3023 in 2019), of which 128 met the clinical and laboratory criteria for UTI, corresponding to a positivity rate of 2.5%. In 2022 and 2023, 2916 cultures were performed (1423 in 2022 and 1493 in 2023), with 75 confirmed cases (2.6%). This represents an approximate 42% reduction in the total number of urine cultures performed, while the positivity rate remained similar between periods. Notably, a substantial proportion of positive urine cultures did not meet the clinical criteria for UTI, highlighting the importance of clinical correlation in case definition (Figure 1).

Regarding demographic characteristics, female patients accounted for 65.6% of cases in 2018-2019 and 49.3% in 2022-2023, with a statistically significant difference between periods (p=0.03). In neonates, males predominated in both periods (66.7 and 55.6%, respectively). Among infants, a shift in sex distribution was observed, with females representing 56.2% in the pre-pandemic period and males 68.4% in the pandemic period. In preschool and school-aged children, female predominance remained stable at 76% in both periods.

**Figure 2.**
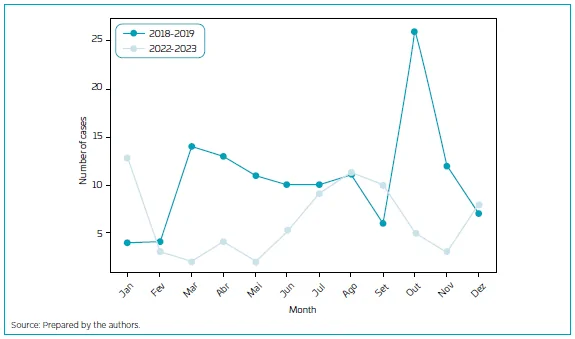
Monthly distribution of confirmed urinary tract infection cases in children during the pre-pandemic (2018-2019) and pandemic (2022-2023) periods.

Seasonal variation in UTI cases was observed across periods. In 2018-2019, peaks occurred in October, March, and April, whereas in 2022-2023, peaks were identified in January, August, and September (Figure 2), suggesting a shift in the seasonal pattern of infections. No formal statistical analysis of seasonality was performed.

Regarding microbiological findings, *E. coli* remained the most frequent pathogen in both periods. A significant decrease in *P. mirabilis* was observed, while *K. pneumoniae* and *P. aeruginosa* showed a significant increase in frequency (p=0.02 for both) (Table 1).

**Table 1.** Epidemiological and microbiological characteristics of children with confirmed urinary tract infection in the pre-pandemic (2018-2019) and pandemic (2022-2023) periods.

Variable	2018-2019 n=128 (%)	2022-2023 n=75 (%)	p-value
Sex			
Female	84 (65.6)	37 (49.3)	0.033*
Male	44 (34.4)	38 (50.7)	
Age group			
Neonates (0-28 days)	2 (1.6)	9 (12.0)	**0.009** ^†^
Infants (29 days-2 years)	65 (50.7)	38 (50.7)	
Preschoolers (2-7 years)	46 (36.0)	16 (21.3)	
School-aged children (7-11 years)	15 (11.7)	12 (16.0)	
Pathogens frequency (proportion)			
Escherichia coli	83 (64.8)	44 (58.6)	0.380*
Proteus mirabilis	20 (15.6)	4 (5.3)	**0.020** ^†^
Klebsiella pneumoniae	7 (5.4)	11 (14.6)	0.020*
Enterococcus faecalis	3 (2.3)	1 (1.3)	0.520^†^
Staphylococcus saprophyticus	2 (1.5)	0 (0.0)	0.390^†^
Pseudomonas aeruginosa	2 (1.5)	10 (13.3)	**0.001** ^†^
Enterobacter aerogenes	1 (0.7)	0 (0.0)	0.630^†^

*p-value associated with the ꭓ^2^ test; ^†^p-value associated with the Fisher’s exact test.

Bold values indicate statistical significance (p<0.05).

Source: Prepared by the authors.

Regarding antimicrobial resistance, *K. pneumoniae* showed a significant increase (p<0.001), while *P. aeruginosa* also showed increased resistance (p=0.01). In contrast, *P. mirabilis* showed a reduction in resistance (p=0.01) (Table 2).

**Table 2.** Comparison of antimicrobial resistance among isolated uropathogens in children with urinary tract infection between the pre-pandemic (2018-2019) and pandemic (2022-2023) periods.

Pathogens	Resistance 2018-2019 n (%) (n=128)	Resistance 2022-2023 n (%) (n=75)	p-value
Escherichia coli	46 (57.5)	34 (47.8)	0.180*
Klebsiella pneumoniae	16 (20.0)	25 (35.2)	<0.001*
Pseudomonas aeruginosa	0 (0.0)	4 (5.6)	**0.010** ^†^
Proteus mirabilis	9 (11.2)	0 (0.0)	**0.010** ^†^
Staphylococcus saprophyticus	0 (0.0)	0 (0.0)	NP
Enterobacter aerogenes	1 (0.8)	0 (0.0)	NP
Enterococcus faecalis	1 (0.8)	0 (0.0)	NP

*p-value associated with the ꭓ^2^ test; ^†^p-value associated with the Fisher’s exact test.

NP: not applicable.

Bold values indicate statistical significance (p<0.05).

Source: Prepared by the authors.

In the antibiotic-specific analysis, amikacin and meropenem maintained high susceptibility rates (>98%) in both periods, with no resistant isolates to amikacin. Cefepime, ceftriaxone, cefuroxime, ciprofloxacin, and gentamicin also maintained consistently high susceptibility (>85%) without significant variation. Amoxicillin-clavulanate showed stable activity (approximately 82%). Nitrofurantoin demonstrated a significant increase in susceptibility, from 81.8 to 100% (p=0.006) (Table 3).

**Table 3.** Antimicrobial susceptibility of uropathogens isolated from children with urinary tract infection in the pre-pandemic (2018-2019) and pandemic (2022-2023) periods.

Antimicrobials	Total samples tested		Sensitive samples % (n=cases)		p-value for susceptibility*
	P1 (n=128)	P2 (n=75)	P1	P2	
Amikacin	116	67	100.0 (116)	100.0 (67)	NP
Amoxicillin-clavulanate	39	58	82.1 (32)	82.8 (48)	1.000
Cefepime	116	72	94.8 (110)	91.7 (66)	0.540
Ceftriaxone	107	62	89.7 (96)	87.1 (54)	0.620
Cefuroxime	114	60	85.1 (97)	90.0 (54)	0.480
Ciprofloxacin	120	63	90.8 (109)	85.7 (54)	0.320
Gentamicin	122	66	91.0 (111)	90.9 (60)	1.000
Meropenem	116	68	100.0 (116)	98.5 (67)	0.370
Nitrofurantoin	44	38	81.8 (36)	100.0 (38)	**0.006**
Norfloxacin	39	61	87.2 (34)	78.7 (48)	0.420
Co-trimoxazole	44	63	65.9 (29)	81.0 (51)	0.110

*ꭓ^2^ test.

P1: period 1; P2: period 2; NP: not applicable.

Bold values indicate statistical significance (p<0.05).

Source: Prepared by the authors.

## DISCUSSION

The main findings of this study indicate changes in the epidemiological and microbiological profile of UTIs in children and adolescents. There was a marked reduction in the total number of urine cultures analyzed, while the positivity rate remained stable. An increase in the proportion of neonates was observed. A significant increase was observed in the frequency of *K. pneumoniae* and *P. aeruginosa*, pathogens often associated with healthcare-related infections and more severe clinical presentations. In contrast, *P. mirabilis* showed a significant reduction between periods, whereas *E. coli* remained the predominant pathogen, with stable antimicrobial susceptibility patterns. Differences were observed in antimicrobial resistance, which increased for *K. pneumoniae* and *P. aeruginosa.*

A marked reduction in the number of urine cultures was observed in the second period, while the positivity rate remained similar. This decrease likely reflects changes in healthcare access and diagnostic practices during the COVID-19 pandemic rather than a true reduction in the incidence of urinary tract infections. Reduced attendance at emergency departments, postponement of non-urgent evaluations, prioritization of acute care, and the reorganization of hospital services may have limited the number of laboratory investigations performed. In this context, increased empirical antibiotic use and fewer diagnostic tests may have contributed to underdiagnosis. Therefore, the lower number of confirmed cases should be interpreted with caution, as it may represent altered healthcare-seeking behavior and diagnostic patterns rather than a genuine epidemiological decline. Similar patterns have been described in other pediatric infectious conditions during the pandemic.^10^ ^,^ ^11^ ^,^ ^12^ ^,^ ^13^

Regarding age and sex, there was a predominance of infants in both periods, reinforcing the vulnerability of this age group due to immunological immaturity and reduced bladder emptying capacity.^5^ The pattern described in the literature indicates a female predominance from infancy onward, attributed to the shorter urethra, the proximity of the urinary meatus to the anal region, and greater perineal colonization, which facilitates ascending infection.^12^ ^,^ ^13^ ^,^ ^14^ However, in the present study, although no statistically significant difference between sexes was observed, a relatively higher proportion of male infants with UTI was identified during the pandemic period (2022-2023). This finding diverges from the expected pattern and may reflect sampling variations or a distinct profile of patients evaluated, with a predominance of more severe and hospitalized cases, in which urine culture collection is more frequently indicated.^15^ The proportion of neonates increased during the second period, and this needs to be addressed. Neonates admitted to the service were born in other institutions, as the hospital does not have a maternity ward. UTIs are a frequent cause of community-acquired late neonatal sepsis and may represent 32% of these infections with a positive blood culture, more frequent than early onset and hospital-acquired neonatal sepsis.^16^ The present study did not investigate blood cultures. Liang et al. investigated a cohort that included children aged under 17 years between 2016 and 2021, and a different definition for UTI, but they did not analyze neonates separately.^13^

Seasonal variation in UTIs differed between periods. While in 2018-2019 peaks were observed in autumn and late summer, in 2022-2023 they were more frequent in summer and late winter. Although the seasonality of pediatric UTIs is not fully understood, factors such as temperature, hydration status, diaper use, and voiding patterns may influence their occurrence.^7^ ^,^ ^17^ Similar findings have been reported for other pediatric infections, reflecting the indirect impact of social distancing measures on the dynamics of infectious diseases in children.^11^ During the pandemic period, changes in healthcare-seeking behavior, school attendance, and environmental exposures may have contributed to this seasonal shift, altering the pattern of case detection.^12^

The microbiological profile showed relevant differences between the periods. *E. coli* remained the predominant etiologic agent in both periods, consistent with its leading role in pediatric UTIs reported in the literature, accounting for 70-90% of community-acquired cases.^14^ However, a significant increase was observed in *K. pneumoniae* and *P. aeruginosa*, along with a reduction in *P. mirabilis*. This change suggests a possible selection of opportunistic and hospital-associated pathogens in the post-pandemic period, a phenomenon also described in studies conducted after 2020.^7^ ^,^ ^17^ ^,^ ^18^ The widespread use of broad-spectrum antibiotics during hospitalizations for COVID-19 and increased exposure to healthcare environments may have contributed to this shift, favoring microorganisms with greater potential resistance and reducing the detection of typical community pathogens. Unfortunately, we did not investigate antibiotic use during these periods for comparison.

An increase in resistance was observed for some species in the second period. This apparent discrepancy can be explained by an ecological effect: the composition of the bacterial population shifted, with a greater participation of intrinsically more resistant pathogens, such as *K. pneumoniae* and *P. aeruginosa*.^19^ ^,^ ^20^ ^,^ ^21^ ^,^ ^22^ The proportional increase of these hospital-associated agents elevates the aggregate resistance rate. Therefore, the rise in global resistance should be interpreted cautiously, as a change in the microbiological landscape toward species with naturally broader resistance mechanisms may have occurred. Also, these pathogens are known to exhibit a higher likelihood of multidrug resistance in similar clinical settings, as reported in other pediatric studies.^18^ ^,^ ^20^ ^,^ ^21^

*Klebsiella pneumoniae* is known for its ability to acquire and disseminate resistance genes, such as those encoding extended-spectrum β-lactamases (ESBLs) and carbapenemases, whereas *P. aeruginosa* exhibits intrinsic resistance to multiple antibiotic classes and remarkable adaptive capacity during treatment.^21^ ^,^ ^22^ These factors partly explain the increase observed and highlight the importance of continuous microbiological surveillance and periodic revision of empirical treatment protocols, especially in hospital settings.^8^ ^,^ ^20^ ^,^ ^21^ ^,^ ^22^

The significant reduction of *P. mirabilis* observed during the pandemic period in this study likely reflects a change in the profile of patients seen and in the predisposing conditions for complicated UTIs. This pathogen is classically associated with infections in children with urinary tract malformations, catheter use, or urinary stones.^22^ ^,^ ^23^ During the pandemic, the suspension of elective procedures and the reduced hospital exposure of patients with chronic urological conditions may have limited these opportunities for infection, explaining the observed decline. The lower isolation rate of *P. mirabilis,* therefore, suggests an indirect impact of healthcare restrictions on the etiological spectrum of pediatric UTIs.

Considering the antimicrobial susceptibility profile, high sensitivity rates were observed for amikacin, meropenem, third- and fourth-generation cephalosporins, ciprofloxacin, and gentamicin. These findings indicate that, despite changes in pathogen distribution, the empirical regimens remained appropriate for the local microbiological scenario, without significant loss of efficacy.^14^

Among the antibiotics evaluated, nitrofurantoin showed a significant increase in sensitivity, reaching 100% in the second period. This finding reinforces its role as a first-line oral option for uncomplicated lower urinary tract infections (cystitis) in pediatric patients,^24^ particularly in outpatient settings. Owing to its excellent urinary concentration and low resistance rates, it remains a safe and effective option for ambulatory management.^25^ ^,^ ^26^ However, it is not suitable for febrile UTIs or suspected upper urinary tract infections due to its limited systemic penetration.^27^

In contrast, hospitalized patients with febrile or complicated UTIs often require parenteral therapy. In this context, ceftriaxone remains an appropriate first-line empirical option, given its maintained susceptibility rates.^14^ Broader-spectrum agents, such as meropenem, should be reserved for severe cases or when multidrug-resistant organisms are suspected.^8^

Amoxicillin-clavulanate also maintained stable susceptibility rates between the analyzed periods. It can be administered orally or intravenously and is indicated for febrile community-acquired UTIs requiring hospitalization, provided there are no severity criteria warranting broader-spectrum agents.^28^ Although the number of isolates tested against amoxicillin-clavulanate was limited, the sustained susceptibility supports its continued role as a step-down or outpatient option, particularly in settings with low prevalence of β-lactamase-producing pathogens.^29^

The increased frequency of *K. pneumoniae* and *P. aeruginosa*, organisms commonly associated with resistance mechanisms such as ESBL production, reinforces the importance of continuous microbiological surveillance and prudent antimicrobial stewardship.^8^ ^,^ ^25^ ^,^ ^30^ The sustained effectiveness of empirical regimens will depend on periodic reassessment of local resistance patterns and rational antibiotic use in hospital settings.

Overall, the findings of this study suggest that the COVID-19 pandemic indirectly influenced the epidemiology and resistance profile of pediatric UTIs, revealing subtle yet relevant changes in the local microbiological landscape. Although overall susceptibility rates remained stable, the higher frequency of hospital-associated pathogens reinforces the importance of strengthening microbiological surveillance and maintaining rational antimicrobial use.

Some limitations should be considered when interpreting the results of this study. The retrospective design, based on laboratory records and secondary data, may have introduced information bias and limited the evaluation of relevant clinical variables, such as comorbidities, previous UTI episodes, and recent antimicrobial use. In addition, microorganisms were not uniformly tested against all antibiotics, which may have affected comparisons between periods. Differences in the number of samples between periods may also influence statistical representativeness. Furthermore, the absence of data on the use of invasive devices, such as urinary catheters, limits the analysis of factors associated with nosocomial infections and multidrug-resistant organisms. As an ecological study, subject to the ecological fallacy, it is essential to understand that it measures aggregate data, and the results refer to the group rather than to individuals. On the other hand, a comparison between two non-consecutive periods of data avoids temporal ambiguity. Finally, this was a single-center study including only hospitalized patients, which may limit the generalizability of the findings.

In conclusion, the COVID-19 pandemic period was associated with changes in the epidemiological and microbiological profile of pediatric UTIs. The increased frequency of healthcare-associated pathogens highlights the importance of continuous microbiological surveillance and rational antimicrobial use in pediatric practice.

## Data Availability

The database that originated the article is available, with a corresponding author.
